# Characterization of Sublingual Microvascular Tortuosity in Steady-State Physiology and Septic Shock

**DOI:** 10.3390/biomedicines13030691

**Published:** 2025-03-11

**Authors:** Athanasios Chalkias, Nikolaos Papagiannakis, Konstantina Katsifa, Antonios Destounis, Athanasios Gravos, Sofia Kanakaki, Georgios Karapiperis, Faidra Koufaki, Athanasios Prekates, Paraskevi Tselioti

**Affiliations:** 1 Institute for Translational Medicine and Therapeutics, University of Pennsylvania Perelman School of Medicine, Philadelphia, PA 19104-5158, USA; 2 OUTCOMES RESEARCH Consortium^®^, Houston, TX 77030, USA; 3 Department of Critical Care Medicine, General Hospital of Piraeus “Tzaneio”, 18536 Piraeus, Greece; 4 First Department of Neurology, Eginition University Hospital, Medical School, National and Kapodistrian University of Athens, 11528 Athens, Greece

**Keywords:** cardiovascular dynamics, circulatory dynamics, hemodynamics, microcirculation, hemodynamic coherence, tissue perfusion, septic shock, oxygen transport, physiology

## Abstract

**Background:** The characteristics of hemodynamic coherence in healthy states and disease remain unknown. Capillary tortuosity is a morphologic variant of microcirculatory vessels, but its effects have generally not been considered in the assessment of tissue perfusion and oxygenation. We investigated the role of sublingual capillary tortuosity in the hemodynamic coherence of anesthetized adult individuals with steady-state physiology (ASA 1) and patients with septic shock requiring emergency abdominal surgery (ASA 4E and 5E). **Methods:** Sublingual macro and microcirculatory variables, oxygen transport, metabolic parameters, and the capillary tortuosity score (CTS) were assessed. **Results:** Mean (SD) CTS was 0.55 (0.76) and 3.31 (0.86) in the steady-state and septic shock group, respectively (*p* < 0.001). In patients with septic shock, CTS was significantly associated with alveolar-to-arterial oxygen gradient (r = 0.658, *p* = 0.015) and oxygen debt (r = −0.769, *p* = 0.002). Significant differences were also observed in Consensus Proportion of Perfused Vessels (PPV; *p* < 0.001), Consensus PPV (small) (*p* < 0.001), Microvascular Flow Index (*p* < 0.001), vessel diameter (*p* < 0.001) and length (*p* < 0.001), wall shear stress (*p* < 0.001), lactate (*p* < 0.001), oxygen extraction ratio (*p* = 0.001), arterial oxygen content (*p* < 0.001), venous oxygen content (*p* < 0.001), oxygen delivery (*p* < 0.001), oxygen consumption (*p* < 0.001), and oxygen debt (*p* = 0.002) between the two groups. **Conclusions:** Sublingual tortuosity was essentially absent in individuals with steady-state physiology. In contrast, it was significantly increased and associated with Alveolar-to-arterial oxygen gradient and oxygen debt in critically ill patients with septic shock.

## 1. Introduction

Hemodynamic coherence is the balance that must be preserved between the macrocirculation and microcirculation to maintain tissue perfusion. Although the term was first mentioned in 1850, its characteristics both in healthy states and disease are largely unknown. This is primarily due to the complex mechanisms required to maintain homeostasis over a very wide range of oxygen concentrations and/or perfusion changes, as well as due to the heterogeneity of critical illness. Most clinicians are currently blind to what is happening in the microcirculation of organs and limit hemodynamic resuscitation to an optimization of systemic hemodynamics [1]. Nevertheless, relying purely on macrohemodynamic targets may fail to improve microvascular perfusion and even prove deleterious.

Videomicroscopy allows for a direct, noninvasive, real-time visualization of capillary networks, facilitating a reliable assessment and an immediate quantitative analysis of the microcirculation at the patient’s bedside. Specifically, direct observation of the sublingual area may provide an excellent window to investigate the microcirculation and a unique insight into the underlying hemodynamic coherence, while the use of an automatic analysis may eliminate observer bias [2]. This is particularly important because sublingual microcirculation reflects visceral microcirculation [3,4] and its assessment may facilitate the implementation of individualized, physiology-guided treatment strategies.

Capillary tortuosity is a morphologic variant of microcirculatory vessels. Although it is not clear how tortuous vessels form, they are thought to result from physiological and pathological phenomena associated with microvascular adaptation including increased cytokine and growth factor signaling, changes in flow dynamics, changes in extracellular matrix composition, decreased mural cell coverage, and in response to endurance training [5,6,7]. It is plausible, though, that the behavior of these vessels plays a central role in processes associated with blood distribution and oxygen transport to tissue. Nevertheless, the effects of microvascular tortuosity are generally not considered as an important hemodynamic quantity in health and disease.

In the present study, we investigated the role of sublingual capillary tortuosity in the hemodynamic coherence of anesthetized individuals with steady-state physiology and patients with septic shock.

## 2. Materials and Methods

This was an ancillary analysis of two prospective cohorts [8,9]. We included patients undergoing elective major non-cardiac surgery and surgical patients with septic shock. The underlying studies were conducted in accordance with the Good Clinical Practice guidelines, the Declaration of Helsinki, and relevant regulatory requirements. The original protocol (NCT03851965) was approved by the UHL Institutional Review Board (IRB no. 60580, 11 December 2018). Informed consent was obtained from all subjects involved in the study or their next-of-kin. This work is reported according to STROCSS criteria [10].

### 2.1. Study Objectives

The primary objective was to characterize sublingual microvascular tortuosity in steady-state physiology and septic shock. Our secondary objective was to investigate the association of sublingual tortuosity with (1) macrohemodynamic, (2) microhemodynamic, and (3) tissue oxygenation parameters.

### 2.2. Patients with Steady-State Physiology

We considered 20 adults fulfilling the following criteria: patients of American Society of Anesthesiologists (ASA) physical status 1; sinus rhythm in electrocardiogram; and no evidence of structural heart disease confirmed by preoperative echocardiography [8]. Before anesthesia induction, all patients received 5 mL kg^−1^ of a balanced crystalloid solution to compensate for preoperative fasting and vasodilation associated with general anesthetics. Anesthesia was induced in the supine position and included midazolam 0.15–0.35 mg kg^−1^, fentanyl 1 μg kg^−1^, ketamine 0.2 mg kg^−1^, propofol 1.5–2 mg kg^−1^, rocuronium 0.6 mg kg^−1^, and a fraction of inspired oxygen of 0.7. All patients were intubated in a standardized fashion [11] and were mechanically ventilated using a lung-protective strategy with a tidal volume of 7 mL kg^−1^, positive end-expiratory pressure of 6–8 cmH_2_O, and plateau pressure < 30 cmH_2_O. General anesthesia was maintained by inhalation of desflurane at an initial 1.0 minimal alveolar concentration. Thereafter, depth of anesthesia was adjusted to maintain Bispectral Index (BIS, Covidien, France) between 40 and 60. Intraoperative fraction of inspired oxygen was also adjusted to maintain an arterial oxygen partial pressure of 80–100 mmHg. Normocapnia, normothermia (37 °C), and normoglycemia were maintained during the perioperative period.

### 2.3. Patients with Septic Shock

We included 13 adult patients with septic shock [ASA 4E and 5E; mean SOFA: 10 (3)] requiring emergency abdominal surgery. Septic shock was defined as circulatory and cellular/metabolic dysfunction that persisted despite adequate fluid resuscitation and required the administration of vasopressors [9,12]. Before the induction of anesthesia, all patients with a central venous pressure (CVP) < 4 mmHg received 7 mL kg^−1^ of a balanced crystalloid solution. Vasopressor dose was titrated to maintain an individualized mean arterial pressure (MAP) level (in all cases > 65 mmHg) based on the patient preadmission levels and tissue perfusion via clinical decision. Anesthesia was induced using regimens that contained fentanyl, ketamine, propofol, rocuronium, and a fraction of inspired oxygen of 0.7, and all patients were intubated using a standardized approach [13,14]. Anesthesia was maintained by inhalation of desflurane at an initial 1.0 minimal alveolar concentration. Thereafter, the depth of anesthesia was then adjusted to maintain BIS between 40 and 60. All patients were ventilated using a protective strategy with tidal volume of 7 mL kg^−1^, positive end-expiratory pressure of 5 cmH_2_O, plateau pressure < 30 cmH_2_O, and driving pressure < 15 cmH_2_O. Normoxemia, normocapnia, normothermia, and normoglycemia were also maintained during the perioperative period.

### 2.4. Measurements

#### 2.4.1. Macrohemodynamics

The radial artery was cannulated and connected to a FloTrac/EV1000 clinical platform (Edwards Life Sciences, Irvine, CA, USA) to directly measure MAP, cardiac output, cardiac index, stroke volume, stroke volume variation, and systemic vascular resistance. The internal jugular vein was cannulated with a triple-lumen central venous catheter that was connected to a pressure transducer to measure CVP and central venous oxygen saturation (ScvO_2_). Mean circulatory filling pressure analog and related values were calculated as previously described [15]. Cardiac power output [CPO = (CO × MAP)/451] and power [Power = CO × (MAP − CVP) × 0.0022] were also calculated; these parameters represent the rate of energy input the systemic vasculature receives from the heart at the level of the aortic root to maintain the perfusion of the vital organs in shock states [16]. Before making study measurements, we confirmed that transducers were correctly leveled and zeroed, while the systems’ dynamic response was confirmed with fast-flush tests. Artifacts were detected and removed when documented, and when measurements were out-of-range or systolic and diastolic pressure were similar or abruptly changed (≥40 mmHg decrease or increase within 2 min before and after measurement).

#### 2.4.2. Sublingual Microcirculation

Sublingual microcirculation was monitored using sidestream dark field (SDF+) imaging (MicroScan; Microvision Medical BV, Amsterdam, The Netherlands), in accordance with the guidelines on the assessment of sublingual microcirculation of the European Society of Intensive Care Medicine [2]. In both groups, microcirculation was assessed 30 min after induction of general anesthesia before surgical incision. In patients with septic shock, microcirculation was assessed after normalization of macrohemodynamics.

Prior to advancing the imaging probe towards the targeted microcirculatory area, excess secretions were gently removed using a sterilized gauze moistened with sterile water maintained at the same temperature as the patient’s sublingual area. The latter was measured with a common digital oral thermometer. After carefully advancing, positioning, and maintaining a gentle contact of the MicroScan lens with the sublingual mucosa, we optimized video quality by optimizing focus, illumination, and contrast. Image stability free of motion, bubbles, and pressure-induced artifacts was achieved due to the patient’s neuromuscular blockade and the stable placement and immobilization of the examiner’s hand on the patient’s headrest. Microcirculation videos were obtained from at least five sites with the probe maintained on the same landmark for the entire duration of each recording.

The process of video acquisition was further mediated by a validated automatic algorithm software [AVA 4.3C (Microvision Medical, Amsterdam, The Netherlands)] to ensure adequate brightness, focus, and stability. Before the sublingual perfusion analysis, all videos were evaluated by two experienced raters blinded to all patient data, according to a modified microcirculation image quality score (MIQS) [17]. The best three videos from each recording were analyzed offline by a blinded investigator and with the AVA4.3C Research Software [2,18].

We analyzed the De Backer score (in mm^−1^; it equals the number of crossings of the capillary web × 21), the Consensus Proportion of Perfused Vessels (Consensus PPV, a ratio of perfused capillaries from all visible capillaries given as a percentage), the Consensus PPV (small) (i.e., the PPV of vessels of a diameter ≤ 25 μm), and the Microvascular Flow Index (MFI). To assess the latter, the screen was divided into four quadrants and a score between 0 and 3 reflecting the average red blood cell (RBC) velocity is given per quadrant. Thereafter, MFI is calculated as the mean MFI averaged over the four quadrants.

Vessel diameter, vessel length, and RBC velocity were determined with the latest version of AVA software using a modified optical flow-based algorithm; the method uses per video frame data to measure the overall velocity per vessel segment. Sublingual tortuosity was assessed with the capillary tortuosity score (CTS), a microvascular score with a good inter- and intra-observer variability, that morphologically assesses microvascular architecture based on the number of twists per capillary existing in the field of view [19]. The number of twists among the majority of the capillaries defines the score and varies from pinhead twists to four twists (Score 0: no twists; Score 1: one twist; Score 2: two twists; Score 3: three twists; Score 4: four twists) [19].

#### 2.4.3. Determination of Capillary Shear Stress

The capillary network (pre-capillary arterioles, capillary bed, post-capillary venules) is characterized by a high degree of structural heterogeneity and an asymmetric and irregular distribution in the vasculature [20,21]. As microvascular flow is controlled by viscous forces, microvascular shear stress, i.e., the tangential force of the flowing blood on the endothelial surface of the blood vessel, was estimated using the force balance equation τ_w_ = (ΔP × d)/4 L, where d and L are the diameter and length of a microvessel, respectively, and ΔP is the pressure difference across the capillary [22]. Based on previous studies from our group [8], a ΔP of 0.1 Pa and 0.02 Pa were used for the steady-state and septic shock group, respectively.

#### 2.4.4. Oxygen Transport and Transitions of Metabolism

Alveolar-to-arterial oxygen gradient (A-a O_2_ gradient), expected A-a O_2_ gradient for age, arterial oxygen content [CaO_2_ = (0.0138 × Hb × SaO_2_) + (0.0031 × PaO_2_)], venous oxygen content [CvO_2_ = (0.0138 × Hb × ScvO_2_) + (0.0031 × PcvO_2_)], venous-arterial oxygen content difference (Cv-aO_2_), oxygen delivery (DO_2_ = CaO_2_ × CO × 10), and oxygen consumption (VO_2_ = C(a–v)O_2_ × CO × 10) were monitored. Oxygen extraction ratio was calculated as the ratio of VO_2_ to DO_2_ using the formula O_2_ER = VO_2_/DO_2_ = (SaO_2_ − ScvO_2_)/SaO_2_.

Microcirculatory oxygen transport was also quantified by calculating convective oxygen flow (Q^C^O_2_) using the equation Q^C^O_2_ = *π*d^2^/4 × V_RBC_ × [Hb] × SO_2_ × C_Hb_, which can be used regardless of the underlying hematocrit [23,24,25,26]. In this equation, d is the microvessel diameter, V_RBC_ is the average RBC velocity, SO_2_ is the hemoglobin oxygen saturation in microvessels, and C_Hb_ is the oxygen binding capacity of hemoglobin (1.34 mL O_2_ gHb^−1^; reduced from the stoichiometrically expected 1.39 by the usual presence of 1–2% methemoglobin plus 1–2% COHb) [23,27,28]. Sublingual SO_2_ was set at 0.47 based on previous studies and the described dynamic balance between oxygen supply and consumption in healthy states and conditions of septic shock [23,29,30,31,32,33,34]. Determination of hemoglobin concentration was based on the volumetric relationship between the RBCs and the plasma, when blood is streaming through microvessels of different diameter (Table 1) [32,34].

Transitions from aerobic to anaerobic metabolism were monitored using oxygen debt (OXD). The latter can be calculated at the bedside using the formula described by Dunham et al., which involves the relationship between lactate and base excess [OXD = 6.322 (Lactate)—2.311 (BE)—9.013] [35] and demonstrates alterations in DO_2_/VO_2_ with a solid physiological basis [36,37]. A negative OXD value indicates normal physiology with aerobic metabolism and adequate physiological reserves, while a positive OXD value indicates an underlying state of oxygen deficiency and anaerobic metabolism.

### 2.5. Statistical Analysis

A statistical analysis was performed using R v4.4 software. Descriptive statistics are presented as mean (standard deviation; SD). The Mann–Whitney test was employed to assess the statistical significance of the differences in CTS between groups. Spearman’s method was used to estimate the strength of the correlations between CTS and the various microcirculation parameters. In order to adjust for the presence of multiple comparisons, the Benjamini–Hochberg false discovery rate correction was utilized [38]. *p*-values less than 0.05 were considered significant.

## 3. Results

Demographic and clinical characteristics of patients are depicted in Table 2 and Table S1 [8,9]. A statistically significant difference was observed in age (*p* < 0.001), sex (*p* = 0.009), ASA score (*p* < 0.001), BMI (*p* = 0.002), and anesthesia parameters between the two groups.

**Figure 1:**
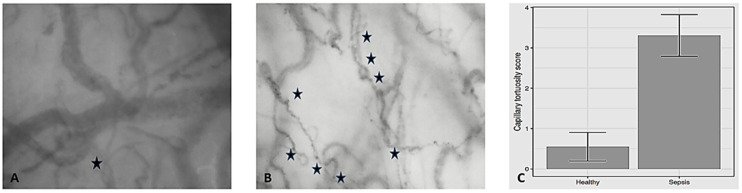
Differences in capillary tortuosity score between steady-state (**A**) and septic shock (**B**). Mean (SD) capillary tortuosity score (**C**) was 0.55 (0.76) vs. 3.31 (0.86), respectively (*p* < 0.001). Each star indicates the presence of capillary twist(s).

Intraoperative hemodynamic variables are presented in Table 3. A significant difference was observed in heart rate (*p* = 0.003), stroke volume (*p* = 0.001), stroke volume variation (*p* < 0.001), systemic vascular resistance (*p* < 0.001), CVP (*p* < 0.001), mean circulatory filling pressure analog (*p* < 0.001), Consensus PPV (*p* < 0.001), Consensus PPV (small) (*p* < 0.001), MFI (*p* < 0.001), vessel diameter (*p* < 0.001), vessel length (*p* < 0.001), τ_w_ (*p* < 0.001), and CTS (*p* < 0.001) between the two groups (Figure 1).

Intraoperative oxygen transport and metabolic variables are presented in Table 4. A significant difference was observed in venous-arterial carbon dioxide difference (*p* < 0.001), bicarbonate (*p* < 0.001), hemoglobin (*p* < 0.001), lactate (*p* < 0.001), A-a O_2_ Gradient (*p* < 0.001), peripheral oxygen saturation (*p* < 0.001), arterial oxygen saturation (*p* < 0.001), ScvO_2_ (*p* = 0.015), O_2_ER (*p* = 0.001), CaO_2_ (*p* < 0.001), CvO_2_ (*p* < 0.001), Cv-aO_2_ (*p* < 0.001), DO_2_ (*p* < 0.001), VO_2_ (*p* < 0.001), Q^C^O_2_ (*p* < 0.001), and OXD (*p* = 0.002) between the two groups.

### 3.1. Capillary Tortuosity in Individuals with Steady-State Physiology

Mean (SD) CTS was 0.55 (0.76). In this group, CTS was significantly associated with DAP (r = –0.471, *p* = 0.036), Consensus PPV (small) (r = –0.458, *p* = 0.042), arterial partial pressure of carbon dioxide (r = 0.512, *p* = 0.021), base deficit (r = −0.463, *p* = 0.04), hemoglobin (r = –0.459, *p* = 0.042), expected A-a O_2_ Gradient for age (r = −0.685, *p* = 0.001), and CaO_2_ (r = –0.474, *p* = 0.035) (Table 5 and Table 6).

### 3.2. Capillary Tortuosity in Patients with Septic Shock

Mean (SD) CTS was 3.31 (0.86). In this group, CTS was significantly associated with A-a O_2_ Gradient (r = 0.658, *p* = 0.015) and OXD (r = –0.769, *p* = 0.002) (Table 7 and Table 8).

### 3.3. Association of Capillary Tortuosity with Hemodynamic and Oxygen Transport/Metabolic Variables in the Entire Study Sample

Spearman’s method was used to estimate the strength of the association between CTS and the assessed hemodynamic and oxygen changes/metabolic variables using the entire study sample (N = 33). Capillary tortuosity score was significantly associated with several hemodynamic and oxygen transport/metabolic variables (Table S2).

## 4. Discussion

To the best of our knowledge, this is the first study to investigate sublingual capillary tortuosity and its role in hemodynamic coherence in anesthetized individuals with steady-state physiology and patients with septic shock. Sublingual CTS was significantly increased in patients with septic shock and associated with A-a O_2_ Gradient and OXD. Significant differences were also observed in several macrohemodynamic and oxygen transport/metabolic variables between the two groups. Patients with septic shock were characterized by significant microvascular impairment and lower τ_w_ compared to those with steady-state physiology.

Tortuous capillaries have been observed in skeletal muscles, myocardium, and other organs of humans and animals [39,40,41,42,43,44,45,46,47,48,49,50,51]. Although their clinical significance remains vague, the dynamics and behavior of blood flowing through these vessels may play a central role in various physiological processes such as embryonic development, tissue oxygenation, muscle contraction, new vessel sprouting, and microvascular blood distribution [43,50]. Clinical observations have also linked tortuous vessels to various pathological conditions (e.g., atherosclerosis, diabetes mellitus, coronary disease), while the evidence for others (e.g., hypertension) are contradictory [49,50,51,52,53].

A crucial aspect of sepsis involves cardiovascular/circulatory dysfunction and increases in oxygen demand. The pathophysiology of impaired oxygen transport and extraction is complex and includes microvascular injury, abnormal distribution of blood flow, and increases in the diffusion gradient for oxygen from the capillaries to the mitochondria. Furthermore, in hypoxic conditions, RBCs lose their ability to release vasodilators and cannot contribute to the autoregulation of microvascular blood flow and DO_2_ [54,55,56]. The aforementioned phenomena contribute to the emergence of two of the most striking manifestations of sepsis, i.e., loss of functional capillary density and microvascular heterogeneity. These structural changes are evident in several tissues and organs including the liver, skeletal muscle, intestinal villi, diaphragm, and the sublingual microcirculation [34,54].

In the present study, sublingual microvascular tortuosity was essentially absent in individuals with steady-state physiology. In contrast, it was significantly increased and associated with A-a O_2_ Gradient and OXD in patients with septic shock. Although the underlying pathogenic mechanisms remain unknown, sepsis-induced mechanical instability and remodeling and the increase in the curvature and intraluminal pressures of collateral vessels bridging adjacent regions may enhance vessel tortuosity [6,51]. Apparently, the negative correlation between OXD and CTS in this group was due to resuscitation efforts. Indeed, all patients were macrohemodynamically optimized prior to assessing the microcirculation, which improved lactate and base excess levels and, therefore, OXD in some patients [57,58].

The low sublingual τ_w_ in patients with septic shock, despite the macrohemodynamic optimization and their increased CTS, is intriguing enough and merits additional discussion and consideration. Shear stress may be lower in tortuous microvessels relative to normal capillaries downstream of altered vessel morphology [6]. It is important to remember, though, that the wall shear stress spatial patterns caused by tortuosity are distinct and must be assessed according to the evaluation region [50]. Our findings, in conjunction with previous studies [6,7,50,59,60,61,62], indicate microvascular tortuosity as an adaptive and compensatory mechanism to improve both the convective delivery to the capillary bed and the diffusive transport from RBCs to mitochondria, maintaining aerobic metabolism in the early stages of sepsis and, presumably, when DO_2_ approaches its critical threshold (maximum O_2_ER). Tortuous vessels may function as collateral channels with lower RBC velocities to reduce the rate of capillary oxygen transport, while enhancing local oxygen diffusion to surrounding tissue [50,63,64,65].

**Figure 2:**
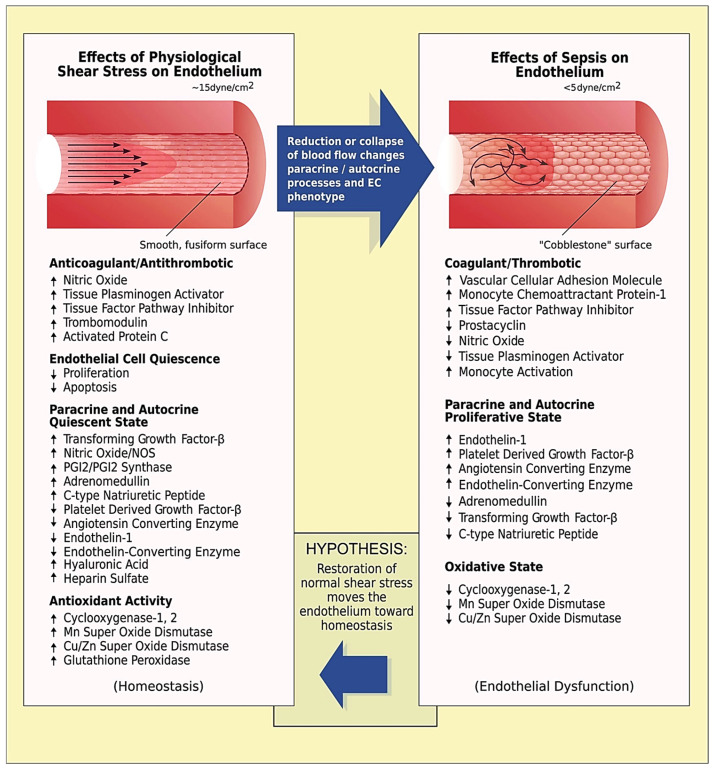
Effects of normal and reduced shear stress on the vessel wall. Left panel: normal endothelium receiving physiological (~15 dynes cm^−2^) shear stress from laminar blood flow patterns. The endothelium exhibits a quiescent fusiform smooth morphology, with increased production of substances that are anticoagulant/antithrombotic and antioxidant. The endocrine status is also summarized, supporting the quiescent status. Right panel: dysfunctional endothelium resulting from low shear stress (<5 dynes cm^−2^). The endothelium displays a hypercoagulant/prothrombotic and pro-oxidant state. Autocrine and paracrine changes listed contribute to this dysfunctional vascular organ. In vitro and in vivo studies suggest that restoration of laminar blood flow and adequate shear stress would move the endothelium towards homeostasis. Reproduced from reference [72] under the terms and conditions of the Creative Commons Attribution (CC BY) license.

In the later stages of sepsis, capillary tortuosity may turn into a maladaptive response depending on the (heterogenous) immune dysregulation and the evolution of critical illness. In patients with severe disease, augmenting tortuosity may increase lumen and wall shear stress [51,66,67,68,69,70,71] in an attempt to compensate for the effects of impaired blood flow on vascular endothelium (Figure 2) [72]. However, this may not be sufficient to prevent the emergence of hemodynamic incoherence (mainly type 1 and/or type 3 microcirculatory alterations) and the exacerbation of the Fåhræus and Fåhræus–Lindqvist effects [8,51,73,74]. Tissue hypoperfusion can be further aggravated by the impairment of RBC membrane deformability and shape recovery; in septic conditions, RBCs become less deformable and more easily aggregate with endothelial cells, thus compromising blood flow [54,75,76,77]. However, since many tissues function physiologically at levels equivalent to an atmosphere of 5% oxygen, and some at levels as low as 1% oxygen [78,79], the role of microvascular tortuosity as a physiological response to sepsis appear to be extremely important and should be clarified in subsequent studies.

### Limitations

A number of limitations must be acknowledged. Although the present physiological study includes a relatively small sample size, data collection and analyses were conducted by blinded investigators, minimizing inter-observer bias and increasing the credibility of study conclusions. Also, we recorded sublingual microcirculation videos from at least five sites and followed the guidelines on the assessment of sublingual microcirculation of the European Society of Intensive Care Medicine [2]. Another limitation is the difference in age and comorbidities between the two groups; thus, the results of the present analysis may be different in other patient populations. In addition, anesthesia can lower the resting metabolic rate and reduce global VO_2_ and has been associated with a reduction in the ability of tissues to extract oxygen. However, we used desflurane for maintenance of anesthesia because it produces mild and stable effects on the microcirculation compared to other agents [8,9]. We also maintained normoxia, normocapnia, normoglycemia, and normothermia to minimize the iatrogenic effects on microvascular perfusion [80,81,82]. All septic shock patients were macrohemodynamically optimized prior to assessing the microcirculation, which improved lactate and base excess levels and, therefore, OXD in some of them. Finally, the pulsatile nature and occasional turbulence of blood flow, the tapered cross-section and distensibility of blood vessels, and the non-Newtonian behavior of blood may limit the accuracy of τ_w_ estimation [83].

## 5. Conclusions

Mean CTS was significantly higher and associated with A-a O_2_ Gradient and OXD in patients with septic shock. While important hemodynamic quantities have been extensively studied over many decades, capillary tortuosity has generally not been considered as a determinant of hemodynamic coherence in health and disease. The present analysis provides interesting insights into the aforementioned relationship. Overall, the increase in sublingual tortuosity appears to be a physiological adaptive response to sepsis-induced microvascular dysfunction and tissue hypoxia. The patterns identified here emphasize the need for a physiological basis for understanding the impact of tortuous morphologies in sepsis and other pathological conditions.

## Figures and Tables

**Table 1:** Reduction in the hematocrit achieved by reducing the microvessel diameter.

Microvessel Diameter (μm)	Blood Volume		Average Velocity of RBCs *
	Microvessel Hct (%)	Plasma (%) (1—Microvessel Hct)	
1100	40.5	59.5	100
750	40.1	59.9	101
450	39.8	60.2	103
250	39.2	60.8	106
95	33.6	66.4	135
50	28.0	72.0	175

* That of plasma = 100. Hct, hematocrit; RBC, red blood cell. The table gives the change in the volumetric relationship between the RBCs and the plasma, when blood from a healthy person is streaming through microvessels of different diameter, and the calculated average velocities of the RBCs in proportion to those of the plasma. With decreasing diameter of the microvessels below 0.1 mm the relative RBC volume is very rapidly decreasing, while the velocity of the RBCs in proportion to that of the plasma very rapidly increasing. Modified from references [32,34].

**Table 2:** Demographic and clinical characteristics of patients.

	Steady-State (n = 20)	Septic Shock (n = 13)	*p*-Value
Age (years)	39.7 (7.06)	70.5 (8.97)	<0.001
Sex (male), n (%)	12 (60%)	9 (69.2%)	0.009
ASA class, n (%)			
1	20 (100)	0 (0)	<0.001
2	0 (0)	0 (0)	NA
3	0 (0)	0 (0)	NA
4	0 (0)	0 (0)	NA
4E	0 (0)	7 (54)	<0.001
5E	0 (0)	6 (46)	<0.001
Height (cm)	176.4 (5.83)	173.85 (5.67)	0.178
Weight (kg)	76.4 (7.78)	81.92 (6.82)	0.06
Body Surface Area (m^2^)	1.93 (0.13)	1.96 (0.1)	0.396
BMI (kg m^−2^)	24.5 (1.52)	27.15 (2.22)	0.002
History, n (%)			
Ischemic heart disease	0 (0)	5 (38.5)	NA
Hypertension	0 (0)	10 (76.9)	NA
Hypercholesterolemia	0 (0)	8 (61.5)	NA
Diabetes	0 (0)	6 (46.2)	NA
Stroke	0 (0)	3 (23.1)	NA
COPD	0 (0)	4 (30.8)	NA
Temperature (°C)	36.72 (0.12)	36.72 (0.49)	0.823
Bispectral index	42.45 (1.47)	50 (5.12)	0.001
Fraction of inspired oxygen	0.31 (0.03)	0.49 (0.16)	<0.001
Tidal volume (ml kg^−1^ IBW)	560 (52.21)	464.62 (55.92)	<0.001
Respiratory rate (min^−1^)	13.7 (1.08)	17.92 (3.52)	<0.001
PEEP (cmH_2_O)	5 (0)	5.23 (1.92)	0.081
ETCO_2_ (mmHg)	36.9 (0.85)	31 (6.11)	0.002
PIP (cmH_2_O)	17.75 (1.29)	20.38 (5.04)	0.217
Plateau pressure (cmH_2_O)	15.95 (1.39)	19.15 (4.41)	0.044

ASA, American Society of Anesthesiologists Classification; NA, non-applicable; BMI, body mass index; PEEP, positive end-expiratory ressure; ETCO_2_, end-tidal carbon dioxide; PIP, peak inspiratory pressure; IBW, ideal body weight. Data are presented as mean (SD) unless stated otherwise.

**Table 3:** Differences in hemodynamic variables between groups.

	Steady-State (n = 20)	Septic Shock (n = 13)	*p*-Value
Heart rate (bpm)	67.5 (6.98)	96.92 (23.5)	<0.001
Systolic arterial pressure (mmHg)	120 (7.43)	116.92 (22.13)	0.863
Diastolic arterial pressure (mmHg)	71.25 (7.41)	66.54 (14.91)	0.595
Mean arterial pressure (mmHg)	88.13 (6.97)	83.62 (16.76)	0.956
Cardiac output (L min^−1^)	5.04 (0.68)	5.48 (1.01)	0.16
Cardiac index (L min^−1^ m^−2^)	2.6 (0.3)	2.75 (0.54)	0.554
Stroke volume (mL beat^−1^)	74.7 (9.57)	61.54 (26.04)	0.001
Stroke volume variation (%)	5.9 (1.83)	12.69 (5.14)	<0.001
Systemic vascular resistance (dynes s cm^−5^)	1306.3 (176.32)	896.31 (247.49)	<0.001
Central venous pressure (mmHg)	7.05 (0.69)	11.31 (4.29)	<0.001
Mean circulatory filling pressure analog (mmHg)	13.06 (0.86)	18.62 (4.61)	<0.001
Cardiac Power Output (W)	0.99 (0.17)	1.01 (0.27)	0.54
Power (W)	0.9 (0.16)	0.87 (0.24)	0.696
De Backer score (mm^−1^)	3.7 (1.17)	3.62 (1.19)	0.754
Consensus PPV (%)	94.15 (5.66)	60.2 (11.3)	<0.001
Consensus PPV (small) (%)	122.89 (146.74)	50.57 (12.64)	<0.001
Microvascular Flow Index (AU)	2.76 (0.25)	1.83 (0.61)	<0.001
Vessel diameter (μm)	10.07 (5.02)	4.35 (1.83)	<0.001
Vessel length (μm)	141 (154.25)	42.54 (15.98)	<0.001
Red blood cell velocity (μm s^−1^)	15.69 (15.02)	13.46 (12.45)	0.519
Wall shear stress (dyne cm^−2^)	3.86 (2.68)	0.72 (0.36)	<0.001
Capillary tortuosity score	0.55 (0.76)	3.31 (0.86)	<0.001

PPV, proportion of perfused vessels; AU = arbitrary units. Data are presented as mean (SD) unless stated otherwise.

**Table 4:** Differences in oxygen transport and metabolic variables between groups.

	Steady-State (n = 20)	Septic Shock (n = 13)	*p*-Value
Venous-arterial carbon dioxide difference (mmHg)	2.8 (0.89)	9 (1.87)	<0.001
pH	7.39 (0.02)	7.32 (0.11)	0.052
Arterial partial pressure of oxygen (mmHg)	92.5 (5.12)	104.31 (38.73)	0.971
Arterial partial pressure of carbon dioxide (mmHg)	39.2 (1.28)	36.85 (6.44)	0.64
Bicarbonate (mmol L^−1^)	25.6 (0.99)	21.19 (6.85)	0.003
Base deficit (mmol L^−1^)	2.08 (0.19)	−0.19 (7.59)	0.183
Hemoglobin (g dL^−1^)	14.06 (0.94)	9.73 (1.83)	<0.001
Glucose (mg dL^−1^)	113.6 (6.21)	119.62 (39.65)	0.338
Lactate (mmol L^−1^)	0.81 (0.15)	3.45 (2.78)	<0.001
A-a O_2_ Gradient (mmHg)	80.33 (25.43)	198.46 (126.17)	<0.001
Expected A-a O_2_ Gradient for age (mmHg)	13.95 (1.77)	21.65 (2.24)	<0.001
Peripheral oxygen saturation (%)	99.6 (0.5)	95.31 (4.01)	0.001
Arterial oxygen saturation (%)	100 (0)	96.77 (3.11)	<0.001
Central venous oxygen saturation (%)	74.15 (2.3)	77.92 (6.12)	0.015
Oxygen extraction ratio (%)	25.85 (2.3)	19.31 (5.59)	0.001
Arterial oxygen content (vol%)	19.7 (1.3)	13.3 (2.41)	<0.001
Venous oxygen content (vol%)	14.73 (1.4)	10.76 (2.27)	<0.001
Venous-arterial oxygen content difference (vol%)	4.96 (0.78)	2.54 (0.74)	<0.001
Oxygen delivery (mL min^−1^)	973.88 (116.23)	724.19 (160.4)	<0.001
Oxygen consumption (mL min^−1^)	247.43 (35.64)	136.45 (41.54)	<0.001
Convective oxygen flow (μm^2^ sec^−1^ kg^−1^)	26.44 (39.96)	1.1 (1.41)	<0.001
Oxygen debt	−8.62 (1.13)	13.23 (29.62)	0.002

A-a, alveolar to arterial. Data are presented as mean (SD) unless stated otherwise.

**Table 5:** Association of capillary tortuosity score with hemodynamic variables in steady-state physiology (n = 20).

	Rho	*p*-Value	Adjusted for Multiple Comparisons
Heart rate (bpm)	0.114	0.633	0.951
Systolic arterial pressure (mmHg)	0.014	0.955	0.998
Diastolic arterial pressure (mmHg)	−0.471	0.036	0.262
Mean arterial pressure (mmHg)	−0.384	0.095	0.449
Cardiac output (L min^−1^)	0.059	0.803	0.992
Cardiac index (L min^−1^ m^−2^)	0.27	0.25	0.618
Stroke volume (mL beat^−1^)	0.031	0.895	0.992
Stroke volume variation (%)	0.274	0.242	0.618
Systemic vascular resistance (dynes s cm^−5^)	−0.266	0.257	0.618
Central venous pressure (mmHg)	0.065	0.785	0.992
Pmca (mmHg)	0.04	0.869	0.992
Cardiac Power Output (W)	0.03	0.9	0.992
Power (W)	−0.006	0.979	0.998
De Backer score (mm^−1^)	0.202	0.393	0.771
Consensus PPV (%)	−0.369	0.11	0.449
Consensus PPV (small) (%)	−0.458	0.042	0.262
Microvascular Flow Index (AU)	0.083	0.726	0.992
Vessel diameter (μm)	0.137	0.565	0.936
Vessel length (μm)	−0.042	0.86	0.992
Red blood cell velocity (μm s^−1^)	−0.031	0.898	0.992
Wall shear stress (dyne cm^−2^)	0.115	0.628	0.942

Pmca, mean circulatory filling pressure analog; PPV, proportion of perfused vessels; AU = arbitrary units.

**Table 6:** Association of capillary tortuosity score with oxygen transport and metabolic variables in steady-state physiology (n = 20).

	Rho	*p*-Value	Adjusted for Multiple Comparisons
Venous-arterial carbon dioxide difference (mmHg)	0.109	0.646	0.951
pH	0.252	0.285	0.629
Arterial partial pressure of oxygen (mmHg)	−0.309	0.184	0.618
Arterial partial pressure of carbon dioxide (mmHg)	0.512	0.021	0.262
Bicarbonate (mmol L^−1^)	0.338	0.145	0.55
Base deficit (mmol L^−1^)	−0.463	0.04	0.262
Hemoglobin (g dL^−1^)	−0.459	0.042	0.262
Glucose (mg dL^−1^)	0.195	0.41	0.775
Lactate (mmol L^−1^)	−0.042	0.86	0.992
A-a O_2_ Gradient (mmHg)	0.011	0.963	0.998
Expected A-a O_2_ Gradient for age (mmHg)	−0.685	0.001	0.046
Peripheral oxygen saturation (%)	−0.283	0.227	0.618
Central venous oxygen saturation (%)	0.025	0.918	0.992
Oxygen extraction ratio (%)	−0.025	0.918	0.992
Arterial oxygen content (vol%)	−0.474	0.035	0.262
Venous oxygen content (vol%)	−0.413	0.07	0.373
Venous-arterial oxygen content difference (vol%)	−0.165	0.486	0.859
Oxygen delivery (mL min^−1^)	−0.146	0.538	0.92
Oxygen consumption (mL min^−1^)	−0.173	0.467	0.853
Convective oxygen flow (μm^2^ sec^−1^ kg^−1^)	0.112	0.637	0.951
Oxygen debt	0.125	0.599	0.951

A-a, alveolar to arterial.

**Table 7:** Association of capillary tortuosity score with hemodynamic variables in septic shock (n = 13).

	Rho	*p*-Value	Adjusted for Multiple Comparisons
Heart rate (bpm)	0.153	0.618	0.978
Systolic arterial pressure (mmHg)	−0.11	0.722	0.978
Diastolic arterial pressure (mmHg)	−0.127	0.679	0.978
Mean arterial pressure (mmHg)	−0.195	0.523	0.978
Cardiac output (L min^−1^)	0.228	0.455	0.978
Cardiac index (L min^−1^ m^−2^)	0.098	0.75	0.978
Stroke volume (mL beat^−1^)	0.203	0.506	0.978
Stroke volume variation (%)	−0.373	0.209	0.978
Systemic vascular resistance (dynes s cm^−5^)	−0.048	0.875	0.978
Central venous pressure (mmHg)	−0.027	0.929	0.978
Pmca (mmHg)	0.07	0.82	0.978
Cardiac Power Output (W)	−0.025	0.937	0.978
Power (W)	−0.13	0.671	0.978
De Backer score (mm^−1^)	0.341	0.254	0.978
Consensus PPV (%)	0.357	0.231	0.978
Consensus PPV (small) (%)	0.355	0.235	0.978
Microvascular Flow Index (AU)	0.08	0.796	0.978
Vessel diameter (μm)	0.091	0.768	0.978
Vessel length (μm)	−0.03	0.922	0.978
Red blood cell velocity (μm s^−1^)	−0.201	0.509	0.978
Wall shear stress (dyne cm^−2^)	0.286	0.343	0.978

Pmca, mean circulatory filling pressure analog; PPV, proportion of perfused vessels; AU = arbitrary units.

**Table 8:** Association of capillary tortuosity score with oxygen transport and metabolic variables in septic shock (n = 13).

	Rho	*p*-Value	Adjusted for Multiple Comparisons
Venous-arterial carbon dioxide difference (mmHg)	−0.14	0.648	0.978
Fraction of inspired oxygen	0.234	0.442	0.978
pH	0.116	0.706	0.978
Arterial partial pressure of oxygen (mmHg)	−0.46	0.114	0.893
Arterial partial pressure of carbon dioxide (mmHg)	0.031	0.921	0.978
Bicarbonate (mmol L^−1^)	0.176	0.566	0.978
Base deficit (mmol L^−1^)	0.701	0.008	0.209
Hemoglobin (g dL^−1^)	0.021	0.945	0.978
Glucose (mg dL^−1^)	−0.218	0.474	0.978
Lactate (mmol L^−1^)	−0.467	0.107	0.893
A-a O_2_ Gradient (mmHg)	0.658	0.015	0.267
Expected A-a O_2_ Gradient for age (mmHg)	−0.079	0.798	0.978
Peripheral oxygen saturation (%)	0.168	0.584	0.978
Arterial oxygen saturation (%)	−0.015	0.96	0.978
Central venous oxygen saturation (%)	0.098	0.75	0.978
Oxygen extraction ratio (%)	0.058	0.851	0.978
Arterial oxygen content (vol%)	−0.03	0.922	0.978
Venous oxygen content (vol%)	−0.085	0.782	0.978
Venous-arterial oxygen content difference (vol%)	0.215	0.48	0.978
Oxygen delivery (mL min^−1^)	0.03	0.922	0.978
Oxygen consumption (mL min^−1^)	0.169	0.58	0.978
Convective oxygen flow (μm^2^ sec^−1^ kg^−1^)	0.197	0.519	0.978
Oxygen debt	−0.769	0.002	0.118

A-a, alveolar to arterial.

## Data Availability

Data will be made available upon request after publication through a collaborative process. Researchers should provide a methodically sound proposal with specific objectives in an approval proposal. Please contact the corresponding author for additional information.

## Supplementary Materials

The following supporting information can be downloaded at: https://www.mdpi.com/article/10.3390/biomedicines13030691/s1, Table S1: Type of surgery and vasopressor use in both groups; Table S2: Association of capillary tortuosity score with demographic, hemodynamic, and metabolic variables in the full study sample.
