# Supplementary material for: Characterization of Sublingual Microvascular Tortuosity in Steady-State Physiology and Septic Shock
Source: Biomedicines. 2025 Mar 11;13(3):691. doi: 10.3390/biomedicines13030691 (PMC11939869; doi:10.3390/biomedicines13030691)
Supplement: Supplementary file 1 [file biomedicines-13-00691-s001.zip › Table S1.pdf]

Table S1. Type of surgery and vasopressor use in both groups.

	**Steady-state (n=20)**	**Septic shock (n=13)**
Gastrointestinal, n (%)	7 (35)	10
Gastrointestinal / Critical limb ischemia, n (%)	0 (0)	1
Gynecological, n (%)	3 (15)	0 (0)
Thyroidectomy, n (%)	2 (10)	0 (0)
Urological, n (%)	8 (40)	1
Liver resection, n (%)	0 (0)	1
Norepinephrine ($\mu g\ kg^{-1}\ min^{-1}$), mean (SD)	0 (0)	0.52 (0.4)
Arginine vasopressin ($IU\ min^{-1}$), mean (SD)	0 (0)	0.0022 (0.006)
Phenylephrine ($\mu g\ kg^{-1}\ min^{-1}$), mean (SD)	0 (0)	0.24 (0.6)
Ephedrine ($\mu g\ kg^{-1}\ min^{-1}$), mean (SD)	0 (0)	0 (0)

Information is from references 8 and 9.
