# Supplementary material for: Characterization of Sublingual Microvascular Tortuosity in Steady-State Physiology and Septic Shock
Source: Biomedicines. 2025 Mar 11;13(3):691. doi: 10.3390/biomedicines13030691 (PMC11939869; doi:10.3390/biomedicines13030691)
Supplement: Supplementary file 1 [file biomedicines-13-00691-s001.zip › Table S2.pdf]

Table S2. Association of Capillary Tortuosity Score with demographic, hemodynamic, and metabolic variables in the full study sample (N=33).

	**Rho**	**p-value**	**Adjustment for multiple comparisons**
Heart rate	0.683	<0.001	<0.001
Systolic arterial pressure	-0.056	0.759	0.773
Diastolic arterial pressure	-0.234	0.19	0.269
Mean arterial pressure	-0.161	0.371	0.445
Cardiac output	0.29	0.101	0.154
Cardiac index	0.212	0.237	0.326
Stroke volume	-0.514	0.002	0.005
Stroke volume variation	0.646	<0.001	<0.001
Systemic vascular resistance	-0.68	<0.001	<0.001
Central venous pressure	0.584	<0.001	0.001
Mean circulatory filling pressure analogue	0.591	<0.001	0.001
Cardiac Power Output	0.129	0.473	0.52
Power	-0.06	0.739	0.767
De Backer score	0.093	0.607	0.645
Consensus PPV	-0.773	<0.001	<0.001
Consensus PPV (small)	-0.787	<0.001	<0.001
Microvascular Flow Index	-0.655	<0.001	<0.001
Vessel diameter	-0.535	0.001	0.003
Vessel length	-0.57	0.001	0.001
Red blood cell velocity	-0.16	0.375	0.445
Wall shear stress (dyne cm^{-2})	-0.451	0.008	0.034
Venous-arterial carbon dioxide difference	0.743	<0.001	<0.001
pH	-0.201	0.262	0.343
Arterial partial pressure of oxygen	-0.143	0.429	0.481
Arterial partial pressure of carbon dioxide	0.092	0.61	0.645
Bicarbonate	-0.371	0.034	0.058
Base deficit	-0.19	0.29	0.371
Hemoglobin	-0.819	<0.001	<0.001
Glucose	-0.16	0.374	0.445
Lactate	0.585	<0.001	0.001
A-a O_2 Gradient	0.738	<0.001	<0.001
Expected A-a O_2 Gradient for age	0.564	0.001	0.002
Peripheral oxygen saturation	-0.545	0.001	0.002
Arterial oxygen saturation	-0.715	<0.001	<0.001
Central venous oxygen saturation	0.354	0.044	0.073
Oxygen extraction ratio	-0.464	0.006	0.012
Arterial oxygen content	-0.824	<0.001	<0.001

Venous oxygen content	-0.678	<0.001	<0.001
Venous-arterial oxygen content difference	-0.695	<0.001	<0.001
Oxygen delivery	-0.584	<0.001	0.001
Oxygen consumption	-0.64	<0.001	<0.001
Convective oxygen flow	-0.558	0.001	0.002
Oxygen debt	0.411	0.018	0.032

PPV, proportion of perfused vessels; A-a, alveolar to arterial.
